# Editorial: Advances in fruit-growing systems as a key factor of successful production

**DOI:** 10.3389/fpls.2024.1434929

**Published:** 2024-06-19

**Authors:** Geza Bujdoso

**Affiliations:** Research Centre of Fruit Growing, Institute of Horticultural Science, Hungarian University of Agricultural and Life Sciences, Gödöllő, Hungary

**Keywords:** cultivar, rootstock, training form, fruit growing system, fruit production, plant experimental development

## Summary

The fruit production industry has recently been faced with many challenges. Examples include climate change, introduction of new fruit-bearing species into production, traits of newly-bred fruit cultivars, innovations in orchard systems, rootstock/scion interactions, effects of fruit-growing technology and growing systems on yield and fruit quality, emergence of new pathogens and pests, as well as birds and mammals in orchards, organic production, fruit quality and compounds, regulatory frameworks, high labor input, and more. Farmers need to have more up-to-date information and answers to these challenges. Modern fruit production is based on an adequate fruit-growing system, supported by many elements that complete this production.

The fruit-growing system is the key factor in fruit production. All aspects of production must fulfill the requirements of the growing system. The aim of the Research Topic titled ‘Advances in Fruit-Growing Systems as a Key Factor of Successful Production’ is to present the latest information on modern innovations, experimental developments, advanced research, and basic research to increase the efficiency of fruit production in the next century. Fruit production is based on biological, phenological, and biochemical processes. Therefore, all participants in the fruit industry must understand the procedures taking place in the background to have successful growth.

Within this research topic, nine manuscripts were published by 54 co-authors covering various topics and fruit species. 

The use of biostimulants is a common practice in the production of pome and stone fruit species, but it is not in practice in the production of Persian walnut (*Juglans regia* L.). Researchers from Hungary examined the responses of nut characteristics and some phenolic compounds of this nut tree species by applying some biostimulants (Wuxal Ascofol, Alga K Plus, and Kondisol) in a bearing orchard. In conclusion, it can be stated that not all applied biostimulants had the same effects on the walnut trees. The effects of the applied materials depended on the spring weather conditions; when the spring was hot, the effects of the applied materials were positive. On the contrary, when the spring was rainy and cold, there were no effects of the applied materials on the host plants.

A Chinese group of researchers studied the effects of different nitrogen application rates on the accumulation of major nutrients and metabolites in wolfberry *(Lycium barbarum* L.) fruits, and published a paper entitled ‘Effects of different nitrogen application rates and picking batches an the nutritional components of Lycium barbarum l. fruits’. The different nitrogen applications significantly affected the compounds of wolfberry. A 20% lower nitrogen application (675 kg ha^-1^) than the usual practice was beneficial for the wolfberry fruit.

Another group of researchers from China examined the swelling agent treatment on the Xiangti” grape variety and the application of electronic nose identification detection in their paper, titled ‘Effect of swelling agent treatment on grape fruit quality and the application of electronic nose identification detection’. The authors recognized that soluble sugars, soluble solids, soluble proteins and vitamin C were significantly increased, and the contents of hexanal, (E)-2-hexenal, and nonanal aldehydes were significantly decreased. They also confirmed, that the electronic nose can be used to detect, whether the grapes have been treated with swelling effects.

It is an interesting topic to have a research about robotics. It is more interesting to do some research about novel harvesting methods for fruit species having small and relatively soft fruit flesh with picking robots. In a study entitled ‘Is this blueberry ripe?’, a blueberry ripeness detection algorithm for picking robots was developed by a Chinese group of researchers, and the authors solved this challenge with a new algorithm that can recognize the ripe blueberries. The paper entitled ‘Apple detection and instance segmentation in natural environments using an improved Mask Scoring R-CNN Model’ is about robotics in apple production. The authors developed a mask-scoring R-CNN method that is very useful for the identification of the apples on the trees.

From a yield perspective, it is important to better understand the metabolomics and genetic background of reproductive bud development in two different variants (edible fig and caprifig) of fig (*Ficus carica* L.). An Italian group of researchers stated in their published paper entitled ‘Metabolomics and genetics of reproductive bud development in Ficus carica var. sativa (edible fig) and in Ficus carica var. caprificus (caprifig): similarities and differences’ that there were different patterns between the two types of figs observed. RNA-sequencing identified 473 downregulated genes, of which 22 were found only in profichi, and 391 up-regulated genes, of which 21 were found only in mammoni, compared to literature data.

To increase the yield in a vineyard with double cropping under a tunnel within one year is a fantastic result. A group of researchers from China provided more data on this research in their published paper entitled ‘Grapevine double cropping: a magic technology’.

Pomegranate (*Punica granatum* L.) is a new fruit species that can be grown in large areas. To preserve fruit quality after mechanical harvesting, it is necessary to detect damaged fruits. A South African – Nigerian group of researchers examined some detection and classification methods using different cameras (SWIR, NVIR), and they stated that there is huge potential in hyperspectral imaging.

In addition to eight original research papers, a review entitled ‘Application of Convolutional Neural Network-Based Detection Methods in Fresh Fruit Production: A Comprehensive Review’ by a Chinese group of researchers was published. The authors created a convolutional neural network-based deep learning detection technology to better protect fresh fruit on the field.

As can be seen from this editorial article, many different topics are covered in this research topic, in line with the aims of the guest editors.

## Author contributions

GB: Writing – original draft, Writing – review & editing.

